# Gene Expression Differences in Prostate Cancers between Young and Old Men

**DOI:** 10.1371/journal.pgen.1006477

**Published:** 2016-12-27

**Authors:** Yuanchun Ding, Huiqing Wu, Charles Warden, Linda Steele, Xueli Liu, M. van Iterson, Xiwei Wu, Rebecca Nelson, Zheng Liu, Yate-Ching Yuan, Susan L. Neuhausen

**Affiliations:** 1 Department of Population Sciences, Beckman Research Institute of City of Hope, Duarte, California, United States of America; 2 Department of Pathology, Beckman Research Institute of City of Hope, Duarte, California, United States of America; 3 Department of Cellular and Molecular Biology, Beckman Research Institute of City of Hope, Duarte, California, United States of America; 4 Department of Molecular Epidemiology, Leiden University Medical Center, Leiden, The Netherlands; Sloan-Kettering Institute, UNITED STATES

## Abstract

Prostate cancer incidence is increasing in younger men. We investigated whether men diagnosed with Gleason 7 (3+4) T2 prostate cancer at younger ages (≤ 45 years, young cohort) had different mRNA and miRNA expression profiles than men diagnosed at older ages (71–74 years, older cohort). We identified differentially expressed genes (DEGs) related to tumor-normal differences between the cohorts. Subsequent pathway analysis of DEGs revealed that the young cohort had significantly more pronounced inflammatory and immune responses to tumor development compared to the older cohort. Further supporting a role of inflammation-induced immune-suppression in the development of early-onset prostate cancer, we observed significant up-regulation of CTLA4 and IDO1/TDO2 pathways in tumors of the young cohort. Moreover, over-expression of *CTLA4* and *IDO1* was significantly associated with biochemical recurrence. Our results provide clues on the mechanisms of tumor development and point to potential biomarkers for early detection and treatment for prostate cancer in young men.

## Author Summary

The incidence of prostate cancer is increasing in young men, and young men are more likely to develop more aggressive prostate cancers than older men. These findings suggest biological differences between prostate cancers that develop in young men and in older men; yet little data and few studies on men diagnosed under age 50 years exist. In this study, we investigated whether men diagnosed with prostate cancer at young ages (≤ age 45 years) had different gene expression profiles than men diagnosed at older ages (71–74 years). We found that inflammatory and immune-related pathways were up-regulated in the young group as compared to the older group, suggesting fundamental differences in tumor development. Moreover, 21% of the young group, compared to 8% of the older group, had biochemical recurrence of prostate cancer–a surprising result given that both groups were diagnosed in early stages of disease (all T2, Gleason 7 (3+4). The recurrence in the young group was associated with over-expression of two genes involved in immune regulation. After validation in a larger dataset, these may provide clues for potential biomarkers to test for monitoring which young patients are likely to progress.

## Introduction

Prostate cancer (PC) is widely recognized as a disease of older men. Only four percent of men diagnosed with PC are younger than 50 years old, and only ten percent are diagnosed under age 55 years[1]. However, PC incidence is increasing in younger men [2], with an increasing proportion having poorly differentiated cancers at diagnosis [3]. Moreover, young men with high-grade PC have worse cancer-specific survival than older men with similar grade and stage PCs [4,5]. These findings suggest biological differences between PCs developing in young men and in older men; and these differences may have implications for early detection and treatment of early-onset PC. Prior literature on the differences in gene expression between early- and late-onset PC is limited. There has been one report of a small study comparing 11 patients diagnosed under age 50 years and 7 patients diagnosed between 57 to 69 years (mean age of 65 years)[6]. Additionally, The Cancer Genome Atlas (TCGA) only has matched tumor and normal data from four patients diagnosed with PC under age 50 years. In this study, we selected tumor and matched normal samples from a relatively common and homogenous tumor subtype of grade T2 (T2a or T2c) and Gleason score 7 (3+4) and compared differences in gene expression between PC that developed in 24 young men (≤ 45 years) and 25 older men (71–74 years). We asked two questions: 1) between the two age groups, are there differences in prostate tumor-induced changes in gene expression that may explain differences in the etiology of early- and late-onset PC?; and 2) do genes that may underlie prostrate tumor properties, such as growth and invasiveness, differ in young men compared to older men?

## Results

### Identification and validation of differentially expressed genes (DEGs)

In this study, we selected tumor samples from a common and homogenous tumor subtype of grade T2 (T2a or T2c) and Gleason score of 7 (3+4) and compared differences in gene expression between PC that developed in young men (≤ 45 years) and in older men (71–74 years). Clinical characteristics of 49 patients and their tumors are shown in Table 1.

**Table 1:** Clinical characteristics of 49 patient samples.

	Total (N = 49)	Old (N = 25)	Young (N = 24)
Age (years)		71–74	38–45
	N (%)	N (%)	N (%)
Pathology stage			
T2a	14 (29)	6 (24)	8 (33)
T2c	35 (71)	19 (76)	16 (67)
Gleason sum			
7 (3+4)	49 (100)	25 (100)	24 (100)
PSA range	1.9–15.4	2.1–15.2	1.9–15.4
PSA group*			
< = 10.0	33 (67)	19 (76)	14 (58)
>10.0	16 (33)	6 (24)	10 (42)
Race/ethnicity			
Whites	43 (88)	22 (88)	21 (88)
African Americans	2 (4)	1 (4)	1 (4)
Hispanics	2 (4)	1 (4)	1 (4)
Asians	2 (4)	1 (4)	1 (4)

* no significant difference in PSA between samples from the two cohorts (Fisher's exact test, p = 0.23)

Gene expression data were generated using the Illumina Human Whole-Genome DASL (cDNA-mediated annealing, selection, extension, and ligation) microarray chips (details in Materials and Methods). After removing batch effects of processing date using the Combat function in the sva package(S1 Fig), we conducted three age-related comparisons using limma (linear models for microarray data analysis; details in Materials and Methods) and identified differentially expressed genes (DEGs) with absolute fold change (|FC|) greater than 1.5 and false discovery rate (FDR) less than 0.25 in each comparison. We first compared tumor-normal gene expression differences between the young (early-onset) and the older (late-onset) cohorts using the age:tissue interaction contrast [(young.tumor − young.normal) − (old.tumor − old.normal)] in limma. We identified 183 DEGs; this contrast may identify genes responding to tumor development (expression changes from normal to tumor) differently in the young cohort compared to the old cohort (age-dependent tumor-normal difference) (S1 File). We then compared differential gene expression between the normal tissue of the young and old groups using the old.normal versus young.normal contrast. We identified 198 DEGs; DEGs from this contrast may reflect expression changes that normally occur with aging (S1 File). Of these DEGs, there were 61 overlapping genes between the two comparisons. Lastly, in the young.tumor versus old.tumor contrast, we identified five DEGs (*ZIC2*, *ZIC5*, *ZNF439*, *USP54*, and *C2)*; this contrast may reflect differences in intrinsic tumor properties between tumors from the two age cohorts. *ZIC2*, *ZIC5*, and *C2* overlap in the age-dependent tumor-normal difference and age-related tumor-tumor comparisons.

**Fig 1:**
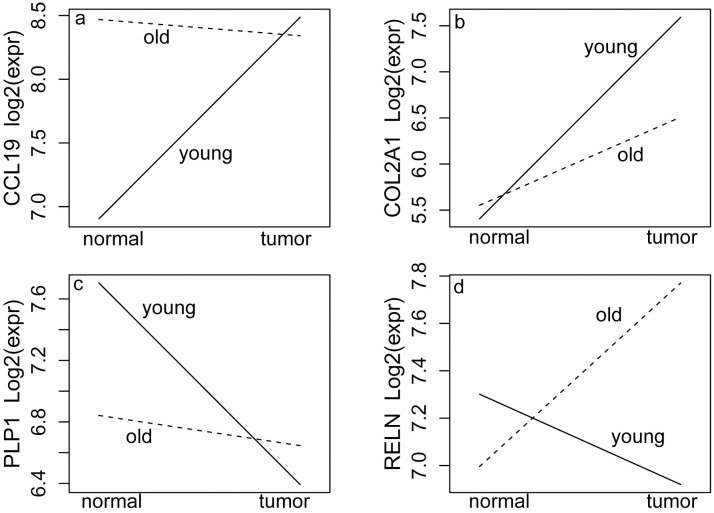
Four main age:tissue interaction patterns for genes that have significant differences in tumor-induced gene expression by age. Horizontal axis is tissue type and vertical axis is mean gene expression. For each interaction pattern, the trend of changes in expression from normal to tumor tissues for the older (dashed line) and young (solid line) cohorts were plotted. There was significantly increased expression in tumor tissue compared to corresponding normal tissue in the young cohort with insignificant change in expression in the older cohort (plot a), whereas in plot b, both cohorts showed increasing expression from normal to tumor with the larger change in the young cohort. In plot c, the young cohort had a significant decrease in expression in tumors compared to the normal tissue, with an insignificant change in the older cohort, whereas in plot d, there was a significant decrease in expression in the young cohort and a significant increase in the older cohort.

Based on the importance of their gene function and expression patterns (representing Fig 1a, 1b and 1c, respectively), we selected three genes (*MMP7*, *COL2A1*, and *SERPINB11*) to validate the DASL assay results. There was a significant correlation (r = -0.81, S2 Fig) between DASL expression values and Delta_Ct (C_t_ target gene – C_t_ reference gene) values from qRT-PCR analysis. We observed over-expression of ERG in tumor samples compared to normal samples. To test whether the over-expression was due to TMPRSS2:ERG fusions, we conducted allele-specific RT-PCR for 49 paired tumor-normal tissue samples. Based on the size variation of PCR products, more than eight types of fusion variants were observed (S3 Fig). Fusion variants, corresponding to over-expression of ERG in the DASL data, were detected in 8 of 25 tumor samples (32%) from the older cohort and 15 of 24 tumor samples (67%) from the early cohort. No fusion variants were detected in normal samples.

### Characterization of DEGs responding differently to tumor development in the two cohorts

We focused on characterizing the 183 DEGs (FDR < 0.25 and |FC| > 1.5) identified from the age:tissue interaction contrast. Of these183 DEGs, 121 genes were up-regulated and 62 were down-regulated in the young cohort compared to the older cohort (S1 File). We observed four basic types of age:tissue interaction patterns having inverse or crossover effects (Fig 1). For each interaction pattern, the direction or magnitude of expression change from tumor to normal samples differed between the two cohorts, indicating that age modified the gene expression changes between tumor and normal samples. For example, type a (Fig 1a) and type b (Fig 1b) interactions were characterized by significant increases of gene expression in tumor compared to normal tissue in the young cohort and non-significant or minimally significant changes of expression in the older cohort. In contrast, type c (Fig 1c) and type d (Fig 1d) interactions were characterized by significant decreases in gene expression in tumor compared to normal tissue in the young cohort and non-significant changes of expression in the older cohort (type c) or significant increases in gene expression in tumors in the older cohort (type d).

The top-five Ingenuity Pathway Analysis (IPA) results based on p-values for the 121 up-regulated DEGs are summarized in Table 2 (S1 File). All top IPA results, including the top pathways enriched in those DEGs, top activated regulators inferred from those DEGs, and top disease involvement of those DEGs, relate to cellular function in inflammatory and immune responses. Seventy of the 121 DEGs are involved with inflammation and immuno-related pathways (S1 File), including 5 genes in the complement family, 12 immune-cell surface antigen genes, 6 chemokine genes, 2 interleukin receptor genes, 2 natural killer cell group genes, and 3 extracellular matrix remodel genes. Furthermore, 57 of the 70 inflammation and immuno-related DEGs demonstrated type a (41 genes) or type b (16 genes) age:tissue interaction patterns with significantly increased expression in tumor compared to normal tissue in the young cohort and non-significant changes in the older cohort. Interestingly, of the 61 DEGs that overlapped between these 183 genes and the DEGs from the old.normal versus young.normal contrast, 38 are immune-related genes, and the pattern was either type a (29 genes) or type b (9 genes). These combined results suggest a significantly more pronounced inflammatory and immune response to tumor development in early-onset prostate cancers than in late-onset prostate cancers. IPA results for the 62 DEGs down-regulated in the young cohort are summarized in S1 Table; 21 of the 62 genes grouped into metabolic pathways. Twenty of the 21 metabolism-related genes (S2 Table) exhibited type c (10 genes) or type d (10 genes) age:tissue interaction patterns(Fig 1), characterized by decreased expression in the young cohort compared to the older cohort.

**Table 2:** Top-five IPA results for the 121 up-regulated DEGs identified from the age:tissue interaction contrast.

**Top Canonical Pathways**	**p-value**	Overlap*
B Cell Development	1.90E-08	17.6% (6/34)
iCOS-iCOSL Signaling in T Helper cells	1.22E-07	7.1% (8/113)
CD28 Signaling in T Helper Cells	2.35E-07	6.5% (8/123)
Primary Immunodeficiency Signaling	2.67E-07	11.5% (6/52)
Calcium-induced T Lymphocyte Apoptosis	1.35E-06	8.8% (6/68)
**Top Upstream Regulators**	**Activation z score**	**Predicted Activation**
TGFB1	2.97	Activated
IL1	2.75	Activated
NFkB(complex)	2.52	Activated
ETS1	2.43	Activated
IL6	2.28	Activated
**Top Diseases and Disorders**	**p-value range**	**Number of Genes**
Inflammatory Response	1.55E-04–2.61E-18	61
Immunological Disease	1.50E-04–4.41E-18	56
Connective Tissue Disorders	1.06E-04–3.66E-15	39
Inflammatory Disease	1.42E-04–3.66E-15	44
Skeletal and Muscular Disorders	7.80E-05–3.66E-15	35

*Overlap: genes shared between 121 DEGs and genes in a canonical pathway.

**Fig 2:**
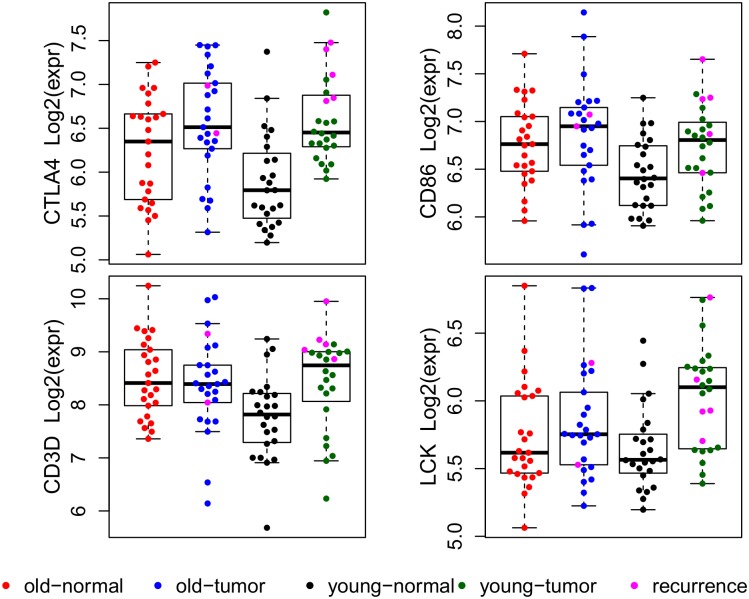
Boxplots and dotplots of four DEGs in the CTLA4 pathway. All four DEGs demonstrate the type b age:tissue interaction pattern with significantly increased expression in tumor compared to normal samples in the young cohort and insignificant expression changes between tumor and normal samples in the older cohort. Patients with biochemical recurrence are shown with a pink color in the corresponding tumor samples.

We ranked the top-five up-regulated gene sets from Gene Set Enrichment Analysis (GSEA) of all 20,261 mRNA genes ordered by t values generated from the age: tissue interaction contrast in limma (Table 3). Over two-thirds of the top-five up-regulated gene sets or pathways from the three GSEA datasets relate to cellular functions in inflammatory and immune responses, consistent with IPA predictions. The CTLA4 pathway was the most significantly up-regulated pathway in the young compared to the older cohort in the GSEA of the BioCarta pathway datasets. The gene expression pattern of four DEGs (*CTLA4*, *CD3D*, *CD86*, and *LCK*) in the CTLA4 pathway for four subgroups of samples categorized by age and tissue status is shown in Fig 2. All four DEGs demonstrated the type b age:tissue interaction pattern (significantly increased expression in tumor compared to normal samples in young cohort and non-significant expression changes between tumor and normal samples in older cohort). The down-regulated gene sets from GSEA ranked based on normalized enrichment score (NES) are listed in S3 Table; more than half are related to metabolic pathways, consistent with IPA results for the 62 of 183 DEGs down-regulated in the young cohort compared to the older cohort.

**Table 3:** Top-five up-regulated gene sets from GSEA of all 20,261 genes ranked by t values generated by the age:tissue interaction contrast.

Top gene sets or pathways ranked by Normalized Enrichment Score (NES)*	Size*	NES	FDR q-value
**BIOCARTA_CTLA4_PATHWAY**	16	2.23	0.00
**BIOCARTA_TOB1_PATHWAY**	15	2.07	0.00
**BIOCARTA_CSK_PATHWAY**	17	2.04	0.01
BIOCARTA_G1_PATHWAY	23	1.97	0.01
BIOCARTA_STATHMIN_PATHWAY	15	1.87	0.02
**KEGG_SYSTEMIC_LUPUS_ERYTHEMATOSUS**	94	2.68	0.00
**KEGG_INTESTINAL_IMMUNE_NETWORK_IGA_PRODUCTION**	33	2.51	0.00
**KEGG_GRAFT_VERSUS_HOST_DISEASE**	27	2.46	0.00
**KEGG_COMPLEMENT_AND_COAGULATION_CASCADES**	45	2.38	0.00
**KEGG_PRIMARY_IMMUNODEFICIENCY**	29	2.35	0.00
**REACTOME_IMMUNOREGULATORY_INTERACTIONS_BETWEEN_ A_LYMPHOID_AND_A_NON_LYMPHOID_CELL**	50	2.62	0.00
REACTOME_GENERATION_SECOND_MESSENGER_MOLECULES	23	2.48	0.00
REACTOME_RNA_POL_I_PROMOTER_OPENING	45	2.34	0.00
**REACTOME_PHOSPHORYLATION_CD3_AND_TCR_ZETA_CHAINS**	15	2.32	0.00
**REACTOME_EXTRACELLULAR_MATRIX_ORGANIZATION**	69	2.32	0.00

*the bold are immune-related gene sets or pathways; Size: the number of genes in each gene set.

### Identification of differentially expressed miRNAs (DEmiRs) and prediction of their regulation on expression of DEGs

Using the age:tissue interaction contrast in limma to analyze miRNA expression data, we identified one DEmiR (has-miR-146b-3p) with FDR < 0.05 and |FC| > 2.0 and 27 DEmiRs with FDR < 0.25 and |FC| > 1.5 (S1 File). From the tumor contrast of the young versus the older cohort, we identified one DEmiR (has-miR-4461) with FDR < 0.05 and |FC| > 1.5 and one additional DEmiR (has-miR-200a-5p) with FDR < 0.25 and |FC| > 1.5.

Because we had miRNA and mRNA expression data for each tumor and matched normal sample, we performed a gene-set global test of association between expression of miRNA and its target genes and further determined the contribution of individual target gene to the miRNA-mRNA association. Based on *in-silico* target prediction and a global association test, 22 of the 27 DEmiRs showed significant expression associations with target DEGs (DEGs from the age:tissue interaction contrast), ranging from 1 target DEG to 57 target DEGs (S1 File). Of the 22 DEmiRs, Hsa-miR146b-5p demonstrated the most significant p value among the global test of DEmiR-DEG associations. Hsa-miR-146b-5p expression had a significant positive correlation (Pearson correlation r > 0.4 and FDR < 0.01) with four target DEGs in inflammation and immune-related pathways (CCR5, CCR7, CXCR4, CD3G). Moreover, down-regulation of 7 of the 22 DEmiRs was significantly associated with increased expression of 19 target DEGs in the inflammation and immune-related pathways.

### Outliers of gene expression in tumor samples

**Fig 3:**
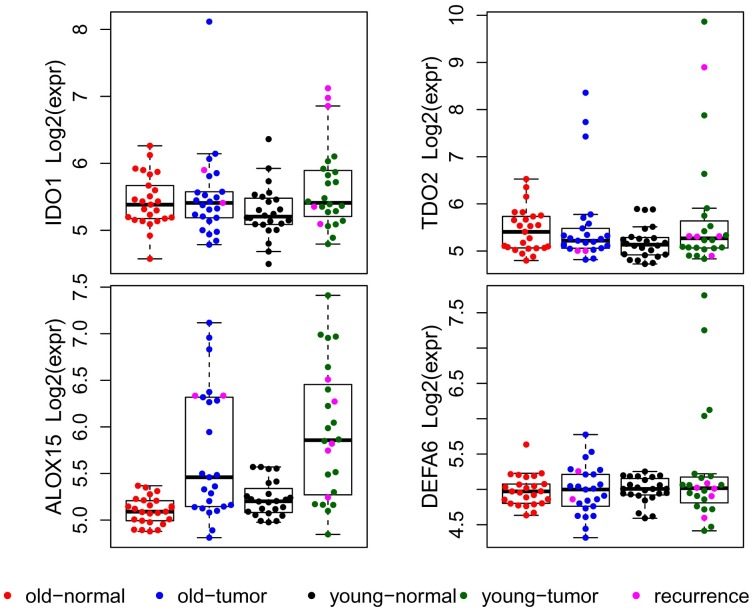
Boxplots and dotplots of DASL data exhibiting outliers of expression in *IDO1*, *TDO2*, *ALOX15* and *DEFA6*. Patients with biochemical recurrence are shown with a pink color in the corresponding tumor samples.

Rare variants may have large effects on gene expression resulting in outliers of expression in those genes in a disease subtype [7]. Because the conventional t-test and ANOVA do not detect rare expression outliers that do not significantly alter the mean within a group, we used the Cancer Outlier Profile Analysis (COPA) [8] method to detect outliers. We found that 79 of 20261 genes showed marked over-expression (outliers) in certain tumor samples (S1 File), including 3 previously reported prostate cancer genes (*ERG*, *ETV1*, *and SPINK1*) [9]. Over-expression of SPINK1 was inversely correlated with over-expression of ERG except in one sample where both were over-expressed (S1 File). IPA was used to investigate whether the 79 genes with outliers of expression shared a common pathway or biological process (S4 Table). Interestingly, the top-five canonical pathways from IPA identified five genes (*IDO1*, *TDO2*, *ALOX15*, *DEFA5* and *DEFA6*) involved in inflammatory and immune responses. DASL expression values for *DEFA5* and *DEFA6* were highly correlated (Pearson correlation r = 0.72). The gene expression patterns for *IDO1*, *TDO2*, *ALOX15* and *DEFA6* in four sample types, classified by tissue and age status, are shown in Fig 3. RNAseq analysis of 11 tumor samples validated the DASL outliers of expression observed in these genes (S4 Fig).

For each gene, outliers were more common in the young than older cohort. Pooling the *IDO*, *TDO2*, *ALOX15*, and *DEFA6* data and using a pathway-based association test, we found significantly more samples with expression outliers in the young cohort than in the older cohort [12 of 24 young patients (50%) compared to 5 of 25 older patients (20%), Fisher’s exact test, p-value = 0.038].

We also examined these four genes in The Cancer Genome Atlas (TCGA) RNAseq data for the 24 patients (≤ 50 years) and 24 patients (70–78 years) (S5 and S6 Tables; S5 Fig). The expression levels between *DEFA5* and *DEFA6* were also highly correlated (Pearson correlation of 0.82). For *TDO2*, *ALOX15*, and *DEFA6*, the outlying expression patterns were similar between the DASL microarray data and TCGA RNAseq data. However, outlying expression of *IDO1* in TCGA was not apparent until we included additional TCGA samples. Conducting the same pathway-based association test in TCGA data for the four genes, we found significantly more samples with expression outliers in the TCGA young-age group than in the older-age group (Fisher’s exact p value of 0.008); 15 of 24 young patients (62.5%) compared to 5 of 24 older patients (20.8%) had at least one expression outlier among the four genes. These results are consistent with our DASL data.

### Characterizing DEGs reflecting difference in tumor properties between the two cohorts

Hierarchical cluster analysis of tumor and normal samples was performed using expression values of the 98 DEGs with unadjusted P value < 0.01 and |FC| > 1.3 identified from the limma contrast of young.tumor versus old.tumor. Forty-six of the 49 normal samples grouped into one cluster with 97% bootstrap support value (S6 Fig); 20 of 24 young cohort tumor samples clustered and 18 of 25 older cohort tumor samples clustered with greater than 80% bootstrap value (S7 Fig). When clustering the 49 tumor samples, the 98 DEGs formed two major vertical clusters, labeled as gene group 1 (51 genes) and gene group 2 (47 genes) (S7 Fig); the “young-cohort tumor” cluster was characterized by co-up-regulation of genes in gene group 1 and co-down-regulation of genes in gene group 2. The “late-onset tumor” cluster had an opposite trend in expression. A similar cluster pattern by age status was observed in TCGA RNAseq data.

From IPA of 98 DEGs, 24 DEGs were involved in construction of tumor morphology, including 9 genes related to extracellular matrix (ECM) remodeling, 3 in cytokine receptor pathways, 3 in Wnt pathway signaling, 1 protease gene, 1 cell adhesion gene, and 1 pro-oncogene (S1 File). Nine of the 24 genes were in the gene group1 clusters and 15 were in gene group 2. Therefore, this opposite trend of co-expression patterns characterizing age-specific cluster of tumor samples may reflect a difference in tumor pathology between early- and late-onset tumors. The gene expression patterns from the DASL data are shown in S8a and S8b Fig. The gene expression patterns for TCGA RNAseq data are shown in S8c and S8d Fig. Using *ARG2* in gene group 1 and *Wnt5A* in gene group 2 as examples, these data indicate no significant expression differences between normal samples from the two cohorts. However, the opposite trend of expression between tumor samples from the two cohorts was observed for the two co-expressed gene groups.

We investigated the association of the gene expression pattern of the 98 DEGs with molecular prostate cancer subtypes ERG-fusion positive (ERG+), non-ERG ETS fusion positive (non-ERG ETS+), over-expression of *SPINK1* (SPINK1+), and triple negative (ERG − / non-ERG ETS ^-^/SPINK1 ^-^) [9,10]. Tumor subtypes for the 49 tumor samples were assigned based on the DASL expression data of *ERG*, *ETS*, and *SPINK1* (S1 File). We then performed supervised hierarchical cluster analysis (S10 Fig). This set of genes did cluster with prostate subtypes; genes in Gene cluster I (right side of image) showed over-expression in the ERG+ tumor subtype and down-regulated expression in SPINK+ tumor subtype and Triple negative tumor subtype; whereas genes in gene cluster II showed opposite expression pattern.

Post-surgery prostate-specific antigen (PSA) data were available for 46 of 49 patients. Of the 46 patients, 7 had biochemical recurrence (defined as a PSA ≥ 0.2 ng/mLwith successive PSA tests ≥ 0.2 ng/mL). Five patients were in the young cohort and two in the older cohort. Over-expression of *IDO1* and *CTLA4* were significantly associated with biochemical recurrence among the five young patients. CTLA4 expression for the 24 early-onset tumor samples had a bimodal distribution pattern with average log2 expression of 7.2 (8 tumor samples) and 6.3 (16 tumor samples) in the high and low-mode groups, respectively (Fig 2); all five young patients with biochemical recurrence were in the high-mode group (p < 0.002, Fisher’s exact test). Similarly, all three young patients with outlying over-expression of IDO1 gene had biochemical recurrence (p < 0.005, Fisher’s exact test) (Fig 3).

## Discussion

In this study, we selected tumor samples from a common and homogenous tumor subtype of grade T2 (T2a or T2c) and Gleason score of 7 (3+4) and compared differences in gene expression between PC that developed in young men (≤ 45 years) and in older men (71–74 years). In this study, we identified 183 DEGs responding differently to tumor development in the young compared to the older cohort. From IPA of 121 of 183 DEGs with up-regulated expression in the young compared to the older cohort, we observed that 70 of the DEGs were enriched in pathways related to cellular function in inflammation and immune responses, indicating a more pronounced inflammatory and immune response to tumor development in early-onset prostate cancers than in late-onset prostate cancers. From GSEA of all 20, 615 genes in our DASL data ranked by t values generated from the age:tissue interaction contrast in limma, the CTLA4 pathway was the most significantly up-regulated pathway in the young compared to the older cohort. Consistent with the DASL data, the CTLA4 and LCK genes in TCGA RNAseq data demonstrated up-regulated expression in tumor compared to matched normal in the young cohort but not in the older cohort (S9 Fig). CTLA4 is an immune checkpoint receptor and up-regulation of the CTLA4 pathway leads to suppression of antitumor immunity [11], which is consistent with the immunosuppression feature found in tumor-associated or tumor-induced inflammation.

Recent studies have illustrated that patient-specific outlying expression in different genes can converge into a unique pathway or related pathways for a disease [12,13]. Using COPA, we identified an additional five DEGs (*IDO1*, *TDO2*, *ALOX15*, *DEFA5* and *DEFA6*) involved in inflammatory and immune responses; outlying expression of these genes was significantly more prevalent in tumors from the young cohort than the older cohort, in both the DASL microarray data and TCGA RNAseq data. Both IDO1 and TDO2 are responsible for degradation of tryptophan, producing a series of catabolites known as kynurenines that regulate immune responses [14]. Similar to CTLA-4, up-regulation of IDO1 or TDO2 allows tumor cells to evade antitumor immunity check from host T cells [15,16].

Up-regulation of immune-related pathways, and especially the pathway involved in immuno-supression, may be a common mechanism related to early-onset cancer development. In a study comparing gene expression patterns between young (<45 years) and older (>65 year) breast cancer cohorts, young-cohort specific gene sets were related to immune function[17]. Similarly, Nam et al [18] re-analyzed microarray data for 12 early-onset colorectal cancer samples and 10 healthy controls using a pathway-based approach and identified two pathways with up-regulation of genes implicated in immuno-suppression, including *CTLA4* and *IDO1*, genes that were also identified in our study. It has been suggested that the balance between tumor growth and destruction of tumor cells by the host immune system can account for the latency of prostate tumors [19]. The tumor cell can modify the tumor antigens resulting in lower immunogenicity and even create an immunosuppressive environment to favor tumor growth. The growing tumor can then trigger a persistent chronic inflammation that further promotes tumor growth. In this study, identification and pathway analysis of DEGs suggest that tumors in young men may have a significant increase in tumor-associated inflammation and an immuno-suppressive microenvironment, which may explain the early initiation and development of detectable tumors in young men.

Mounting evidence suggests that up-regulation of miRNA-146a and miRNA-146b play important roles in the resolution or termination of acute inflammatory responses after a pathogen has been cleared [20–22]. In our miRNASeq data, both miRNA-146a and miRNA-146b demonstrated significantly increased expression in tumor compared to normal tissue in the young cohort with no significant change in the older cohort. Hsa-miR146b-5p showed the most significant p-value among the global test of association between 27 DEmiRs and 183 DEGs. However, its target genes in inflammation and immune-related pathways, such as *CCR5*, *CCR7*, *CXCR4*, and *CD3G*, did not show down-regulated expression in tumor compared to normal samples. One explanation is that tumor-induced inflammation cannot be successfully resolved because it is persistent chronic inflammation [23].

By focusing on Gleason score 7 (3+4) and T2 tumors and matching on ethnicity (Table 1), we identified DEGs reflecting differences in intrinsic tumor properties between tumor samples from the two age groups. From hierarchical clustering analysis, the 98 DEGs identified from the young and older cohort tumor comparison split into two co-expressed gene groups with an opposite trend of co-expression direction in early- and late-onset specific tumor clusters. This opposite trend of co-expression patterns that characterize the age-specific tumor groups may reflect differences in tumor morphology. Expression of *Wnt5A* can activate a non-canonical Wnt pathway and suppress the signal of the canonical Wnt pathway, resulting in decreased proliferation, migration, invasiveness, and clonogenicity of cells, therefore behaving as a tumor suppressor [24]. In our DASL data (S8a Fig and TCGA RNAseq data (S8c Fig), higher expression levels of *Wnt5A* were observed in normal samples compared to tumor samples, consistent with a tumor suppressor role of this gene. Moreover, a significantly lower expression of *Wnt5A* was observed in tumor samples from the young than the older cohort. An additional four genes involved in construction of tumor morphology were also reported to act as tumor suppressors, including *RELN* (serine protease degrading ECM)[25], *HS3ST1* (ECM remodeling gene)[26], *PCDH17* (cell adhesion gene)[27], and *ANGPTL4* (matrix-associated gene)[28]; these four genes shared similar expression patterns as *Wnt5A*. In contrast, 3 of the 24 genes (*ARG2*, *COL2A1*, *FMOD*) showed significantly higher expression in tumor samples than in normal samples in both the DASL and TCGA data. Zhang et al [29] reported that *ARG2* over-expression was associated with migratory and invasive properties of prostate tumor. Bu et al [30] provided evidence that increased expression of *ARG2* was an early event in prostate cancer development, and urine *ARG2/PSA* transcript ratio outperformed serum PSA in diagnosis of prostate cancer. In both our DASL data (S8b Fig) and TCGA RNAseq data (S8d Fig), there was significantly higher expression of *ARG2* in tumor samples in the young than the older cohort, which may suggest a stronger oncogenic role of ARG2 in the development of prostate cancer in young men. Both COL2A and FMOD (a COL2A1 interacting protein) have been reported as biomarkers for prostate cancer (47, 48). *COL2A1* and *FMOD* showed very similar expression patterns as *ARG2* across sample groups defined by tissue and age status, demonstrating a higher expression level in the young tumor group than in the old tumor group. Together, significantly lower expression of tumor suppressor genes and higher expression of oncogenic genes in tumor samples from the young cohort as compared to the older cohort may suggest more invasive tumor properties of early-onset prostate cancer than late-onset prostate cancer.

The study has several limitations. First, although the sample size of matched tumor and normal tissues in the young cohort in our study is larger than prior studies, it is still small. Additional sample sizes are required to confirm whether over-expression of *IDO1* and/or *CTLA4* in tumors may be predictive of prostate cancer recurrence in young men. The molecular tumor subtypes of the 49 samples were consistent with other reports of tumor subtypes: 1) we identified tumor subtypes based on expression of *ERG*, *ETV1* and *SPINK1*, with the same inverse correlation of *ERG/ETV1* expression to *SPINK1* expression; and 2) the prevalence of ERG fusion events was higher in the young cohort compared to the older cohort [6,8,9,31,32]. Second, in this study, we did not have normal tissues from non-prostatectomy specimens. It is possible that normal tissue in the prostate of men with prostate cancer has undergone some genetic changes in response to tumor development. However, the normal tissue expression effects by age were consistent with reports from two independent large studies of aging [33,34], where up-regulation of genes and pathways involved in inflammation and immune responses was a common signature of aging. Lastly, we did not perform functional studies of the effects of these DEGs on actual tumor properties. We hope that this exploratory study will stimulate some new thinking in this field.

In conclusion, even when matching on Gleason score and tumor grade, there are differences in gene expression in prostate tumors from young and older men. It may be that these younger men have less indolent disease, and if not caught early, would have progressed to a higher grade and Gleason score in several years. Moreover, a number of these differences may reflect why prostate cancer diagnosed in younger men is often more aggressive than in older men. We have identified genes and associated pathways that may explain some of the age differences, and that may provide urologists with important information to treat the increasing number of young men with prostate cancers.

## Materials and Methods

### Ethics statement

This study was approved by the City of Hope Institutional Review Board (IRB07244). The study is currently approved through 06/02/2017 with the protocol approved for a Waiver of Informed Consent and Waiver of HIPAA Authorization. There is a waiver of informed consent because the samples are leftover/discard from standard of care procedures. An honest broker process double checks the system to make sure that no specimens are from individuals who dissented for use of their specimens for research studies.

### Patients, tissue samples, and total RNA extraction

Leftover surgical tissue from prostatectomy of 49 patients, including 24 men diagnosed between ages 38 and 45 years and 25 men diagnosed between ages 71–74 years, with tumor surgical stage 2 (2a or 2c) and Gleason score 7 (3+4) were included in the study (Table 1). Follow-up data was available through the California Cancer Registry for all patients and post-surgery PSA test results were available for all but three patients. Mean follow-up times from date of surgery were 76.7 months (range from 17.8 to 158.4 months) and 82.7 months (range from 50.2 to 203.8) for the young and older cohorts, respectively. Tissue samples of primary tumor and matched normal tissues were obtained from formalin-fixed paraffin-embedded (FFPE) tissue blocks isolated from prostatectomies performed between 1998 and 2011 at the City of Hope National Medical Center. A pathologist examined all hematoxylin and eosin (H&E)-stained slides to confirm Gleason score 7 (grades 3+4); samples with tertiary Gleason grade of 4 or 5 were excluded. The area(s) having > 80% epithelial tumor cells or > 90% normal-appearing epithelial prostate cells were circled to identify the regions on the block to be used for tumor and normal core samples, respectively. Total RNA was extracted from approximately 5 mg of unsectioned FFPE core samples using RecoverAll^™^ Total Nucleic Acid Isolation kit (Life Technology Inc.).

### mRNA and miRNA profiling

#### mRNA profile

The Illumina Human Whole-Genome DASL (cDNA-mediated annealing, selection, extension, and ligation) HT Assay was used for mRNA expression profiling of 29,000 genes in the human genome. A tumor-normal sample pair was always on the same chip and samples from the two age groups were evenly distributed on each chip. Using Principal Component Analysis (PCA), 2 of 24 samples in batch 1 and 1 of 24 samples in batch 2 were obvious outliers. New RNA samples for those three pairs of samples were prepared and included in the third batch. No obvious outliers were observed among 56 samples in batch 3. An inter-chip normalization using the quantile normalization method[35] was performed for the 98 samples, including 20 from batch 1, 22 from batch 2, and 56 samples from batch 3. PCA of normalized data revealed the batch-specific sample clusters (S1a Fig). The Combat function in sva package[36] was used to adjust batch effects across the three sample processing batches; it considered both the tissue type factor and age cohort factor as covariates in the model matrix and performed parametric empirical Baysian adjustments. The PCA diagrams before and after batch correction clearly showed the minimizing of batch effect(S1a Fig), retaining the predominant difference between tumor and matched normal tissues (S1b Fig) and indiscernible change of variation associated with the age factor (S1c Fig).

#### miRNA profiling

Small RNA sequencing was performed for the same 98 samples; 24 tumor-normal pairs were sequenced in a first batch and the remaining 25 pairs in second batch; samples from the two age groups were evenly distributed in each batch. 500ng of total RNA was used for small RNA sequence library construction following the Illumina TruSeq Small RNA sample preparation protocol. Samples were sequenced on the Illumina HiSeq2500, and 10 to 15 million reads per sample were generated. Reads mapping and counts per million (CPM) calculations were described previously [37]. Normalization of miRNA data was performed using the trimmed mean of M-value (TMM) method in the edgeR package. Combat was used to remove batch effect as samples were clustered into two groups corresponding to the two sequencing batches (S1d Fig). Similar to DASL array results, Combat successfully minimized the batch effect and did not change the variation associated with tissue type and age group factors (S1d–S1f Fig).

### Statistical analysis of mRNA and miRNA data

#### Identifying DEGs as common biomarkers

DEGs were identified using a mixed linear model with one random effect implemented in limma. In the model, tissue type with two levels (tumor and normal) and age group with two levels (young and old) were considered as categorical variables with fixed effects, and sample ID (normal-tumor pair ID for each patient) was treated as a random effect. The *duplicationCorrelation* function in limma [38] estimated the correlation between gene expression measurements made on the same patient using sample ID as a blocking variable. Five comparison contrasts were extracted from limma: 1) a normal versus normal contrast between the two age cohorts (old.normal − young normal) was used to identify expression changes that normally occur with aging; 2) a tumor versus normal contrast (young.tumor − young.normal) within paired samples from young patients to identify tumor-induced expression changes in the young group; 3) a tumor versus normal contrast (old.tumor − old.normal) in the older group; 4) an age-tissue interaction contrast [(young.tumor − young.normal) − (old.tumor − old.normal)] to identify differences in tumor-induced changes in the young cohort compared to the old cohort (age-dependent tumor-normal difference); and 5) because tumors from the two age cohorts were matched on tumor stage, Gleason score, and patients’ ethnicity, a tumor versus tumor comparison (young.tumor − old.tumor) to identify differences in intrinsic tumor expression between the two age cohorts. Probability values were adjusted for multiple comparisons using the False Discovery Rate (FDR) method of Benjamini and Hochberg [39]. The same analyses were used to identify differentially expressed miRNAs (DEmiRs).

#### Detecting genes with outliers of expression in tumors

The t-test and ANOVA compare difference in mean between sample groups and are often not able to detect aberrant expression as a rare event in tumor group compared to normal group; too few samples with outlying expression in a group may not significantly change the mean difference between two compared groups. Using COPA, Tomlins et al [32] identified three genes (*ERG*, *ETV1* and *SPINK1*) with outlying expression only in a small subset of prostate tumor samples that were not detected by the t-test or ANOVA. Outlying expression of *ERG* and *ETV1* are caused by gene fusion, but outlying expression of *SPINK1* is not [32]. Therefore, COPA is a complement to the conventional t-test and ANOVA when applied to data with within-group heterogeneity. We used COPA [8] to test whether specific genes had outliers of expression from a small number of tumor samples. Ingenuity Pathway Analysis (IPA, Qiagen) was used to explore if those genes with outlying expression disrupt the same pathway. The Fisher’s exact test was used to test the associations between outlying expression (yes or no) in genes within the same pathway and age group (old and young).

### Bioinformatics analyses

#### Pathway analysis by IPA and GSEA

IPA of DEGs was used to predict significant directional effects of DEGs on cellular function and diseases. In contrast to IPA in which a subset of DEGs are selected and analyzed, the GSEA [40] input is a list of all assayed 20,261 genes ranked by t-values generated by the limma analysis. GSEA was conducted for three C2-curated canonical pathway datasets including BioCarta (217 gene sets), KEGG (186 gene sets), and Reactome (674 gene sets).

#### Analysis of potential miRNA regulation of mRNA expression

Integrated analysis of miRNA-mRNA expression regulation [41] was used to investigate possible interactions between DEmiRs and target DEGs. Briefly, for each DEmiR, a set of target DEGs with prediction scores was generated based on *in silico* target predictions from TargetScan, PITA, and microcosm (formerly miRBase Targets). A gene-set global test was used to test association between miRNA expression and its multiple mRNA target genes and further determine the contribution of individual target mRNA genes to the miRNA-mRNA association.

#### Hierarchical clustering analysis

Partek Genomics Suite (Partek, Inc., St. Louis, MO) was used to perform hierarchical clustering analysis with Pearson correlation coefficient as a distance metric and average linkage to measure closeness between two clusters. Pvclust[42] was used to assess uncertainty in hierarchical clustering by calculating approximately unbiased p-value for each cluster based on the strategy of multi-scale bootstrap re-sampling with 10,000 bootstrap replications.

### Validation of DASL-data DEGs by RT-PCR

Three DEGs (*MMP7*, *COL2A1*, and *SERPINB11*) were selected to validate DASL expression data by RT-PCR. HPRT1 was used as a control gene as it showed stable expression in our DASL data and has been reported as the most stable gene among 16 potential candidate references genes in a qRT-PCR study of PC tissues[43]. Appropriate PCR primers, with no significant primer dimer peaks detected in PCR melting curves and spanning exon-exon junctions were designed to amplify PCR products less than 150 bp. High Capacity cDNA Reverse Transcription Kits (Life Technologies) were used for cDNA synthesis, and Power Sybr Green master mix (Life Technologies) was used to run qRT-PCR on an ABI 7900 Realtime PCR system (Life Technologies). Pearson correlations between the ΔCt (Ct of target gene – Ct of housekeeping gene) and microarray expression values were calculated using the cor.test function in R[44].

Allele-specific RT-PCR amplification of eight TMPRSS2:ERG fusion variants reported by Wang et al[45] was carried out to test the correlation between detection of fusion variants and over-expression of *ERG* in the microarray data. ZymoTaq master mix was used for PCR amplification and PCR products were resolved on 2% agarose gel to visualize sizes of fusion variants.

### Validation of DASL outliers of gene expression from COPA using RNA-seq

Outliers of gene expression were defined as genes having a robust-Z-score transformation of DASL data at least three times greater than the Median Absolute Deviation (MAD). RNAseq was performed on 11 of 49 tumor samples exhibiting outliers of gene expression and for whom high quality RNA could be isolated. 500 ng of total RNA was used for paired-end sequencing on the Illumina HighSeq 2500. Reads mapping and reads per kilobase million (RPKM) calculation were described before [46]. RPKM was used for validation of outlying gene expression in DASL data.

### Validation of mRNA expression results using TCGA data

miRNA and RNA-seq prostate adenocarcinoma (PRAD) data were downloaded from TCGA[10]. These data were available for 85 PC patients diagnosed between ages 44 to 50 years (young group) and between ages 70 to 78 years (old group). Because paired normal samples were available only for four patients in the young group and six in the old group, we only compared gene expression differences between tumor samples from the two age groups. Compared to the young group, more prostate tumors in the old group had tumor pathology stage of T3 and high Gleason scores of 8 or 9. Therefore, we matched on pathology stage and Gleason score from the two age groups (procedure described in S5 Table) and identified 24 samples in each age group (see S6 table for clinical characteristics of those selected samples). Batch-effect-removed mRNA sequence data were downloaded using TCGA MBatch web tool [47]. A two-sample t-test was performed to identify DEGs between the two tumor age groups. In addition, we specifically looked at expression levels of genes with outlying expression identified from COPA. In order to analyze expression changes from paired normal tissue for genes in the CTLA4 pathway in TCGA data, we relaxed the age criteria to be older than 65 years (18 patients) and younger than 55 years (14 patients) to increase the sample size.

## Supporting Information

S1 Fig PCA diagram before and after batch correction for batch factor, tissue factor and age factor. (TIFF)

S2 Fig Correlation between DASL microarray expression data (on y-axis) and qPCR expression data (on x-axis) for *MMP7*, *COL2A1*, and *SERPINB11*. (TIF)

S3 Fig RT-PCR of TEMPRSS2-ERG fusion variants (type 1 to type 8) from RNA extracted from formalin-fixed paraffin-embedded (FFPE) prostate cancer tissue blocks. The type 1 variant is T1G2, indicating that exon1 of the TMPRSS2 gene (5’ of fused gene) is fused to exon2 of the ERG gene. Based on the same naming logic, the other seven variants are type 2(T1G3), type 3 (T1G4), type 4 (T1G5), type 5 (T2G2), type 6 (T2G4), type 7 (T2G5), and type 8 (T3G4). For each tissue sample, a separate RT-PCR was performed to amplify each variant. Negative control samples are denoted as C. Ladder was Biorad 20-bp DNA ladder.
(TIF)

S4 Fig Validation of outlying expression of genes from DASL data using RNAseq Eleven samples were selected for RNAseq based on outlying expression in specific genes from DASL expression data. As examples, this figure displays 10 samples with outlying expression validated by RNAseq, including one sample for *IDO1*, one sample for *TDO2*, five samples for *ALOX15*, and three samples for *DEFA6*.
(TIF)

S5 Fig Boxplots and dotplots of RNAseq data from TCGA for *IDO1*, *TDO2*, *ALOX15*, and *DEFA6*. (TIF)

S6 Fig Hierarchical clustering of 49 COH tumor and matched normal samples using gene expression data for the 98 differentially expressed genes (DEGs) identified from the limma analysis comparing tumors from the young and older cohorts. In clusters, columns are samples and rows are the genes. In the heat map, red corresponds to high expression, blue corresponds to low expression, and green corresponds to intermediate expression levels.
(TIF)

S7 Fig Hierarchical clustering of 49 COH tumor samples using gene expression data for the 98 differentially expressed genes (DEGs) identified from the limma analysis comparing tumors from the young and older cohorts. In clusters, columns are samples and rows are the genes. In the heat map, red corresponds to high expression, blue corresponds to low expression, and green corresponds to intermediate expression levels.
(TIF)

S8 Fig Boxplots and dotplots of DASL data for *WNT5A* (plot a) and *AGR2* (plot b) and RNAseq data from TCGA for *WNT5A* (plot c) and *AGR2* (plot d) identified from comparing early-onset tumors (young group) versus late-onset tumors (older group) in limma. (TIF)

S9 Fig Boxplots and dotplots of four DEGs in the CTLA4 pathway in TCGA data set. For TCGA data, mRNA expression data from paired normal tissue were available only for four young prostate cancer patients (≤ 50 years). Therefore, in order to check expression changes for genes in the CTLA4 pathway, we relaxed the age criteria to be older than 65 years (18 patients) and younger than 55 years (14 patients). This is consistent with the expression pattern in the DASL data for the CTLA4 and LCK genes (see Fig 1).
(TIF)

S10 Fig Association between molecular prostate cancer subtypes and expression of the 98 DEGs (|FC|> 1.3 and p < 0.01, identified from the young.tumor versus old.tumor comparison). Tumor subtypes for the 49 tumor samples were assigned based on the DASL expression data of *ERG*, *ETS*, and *SPINK1*. Supervised hierarchical cluster analysis indicated that this set of genes cluster with known prostate subtypes.
(TIF)

S1 Table Top-five Ingenuity Pathway Analysis (IPA) results for 62 of 183 DEGs (down-regulated in young compared to older cohort) from the age:tissue interaction contrast. (DOCX)

S2 Table 21 of 62 down-regulated DEGs from age:tissue interaction contrast classified in the metabolic pathways. (DOCX)

S3 Table Gene Set Enrichment Analysis (GSEA) of down-regulated gene sets or pathways enriched in age-related differentially expressed genes in young compared to older cohorts. (DOCX)

S4 Table Top-five IPA results for 79 genes with outliers of expression identified by the Cancer Outlier Profile Analysis (COPA). (DOCX)

S5 Table A flow diagram for selection of samples from TCGA. (DOCX)

S6 Table Clinical characteristics of 48 TCGA patient samples. (DOCX)

S1 File An excel supplementary file includes supplementary tables longer than one page. (XLSX)
