# Supplementary figures and images for: Gene Expression Differences in Prostate Cancers between Young and Old Men
Source: PLoS Genet. 2016 Dec 27;12(12):e1006477. doi: 10.1371/journal.pgen.1006477 (PMC5189936; doi:10.1371/journal.pgen.1006477)

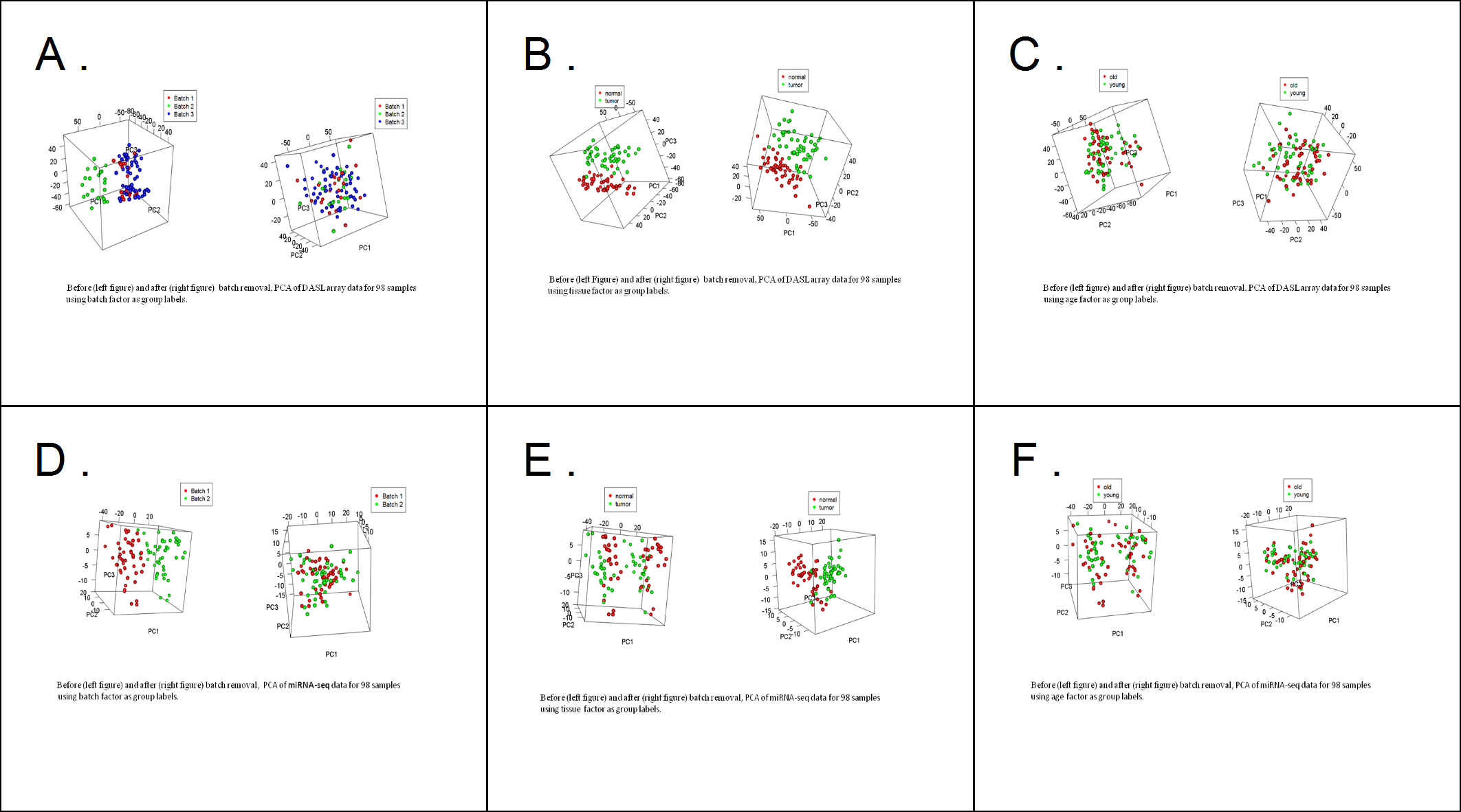

Supplement: S1 Fig — (TIFF) [file pgen.1006477.s001.tiff]

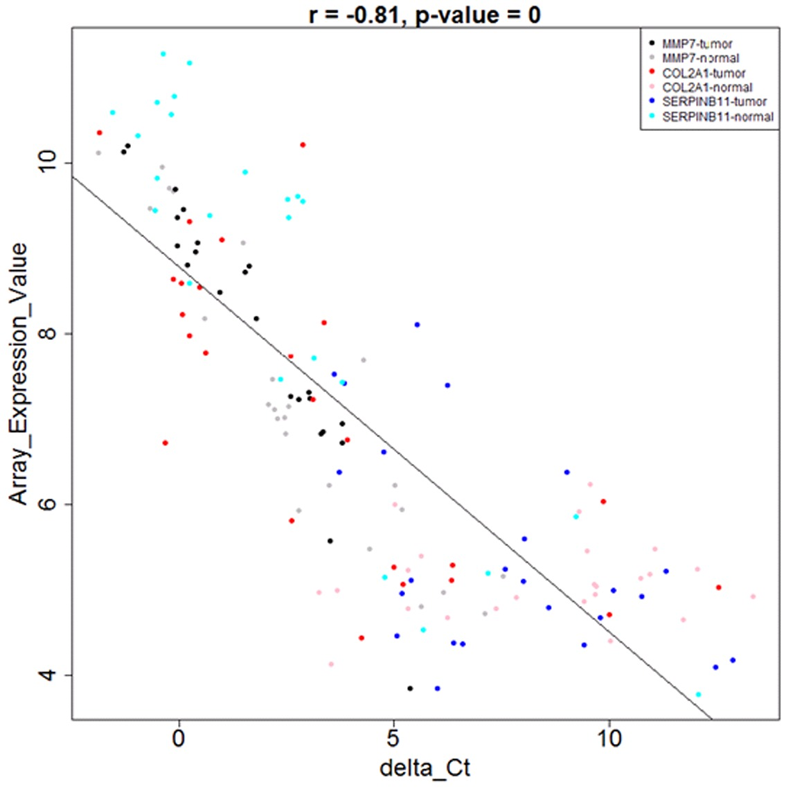

Supplement: S2 Fig — (TIF) [file pgen.1006477.s002.tif]

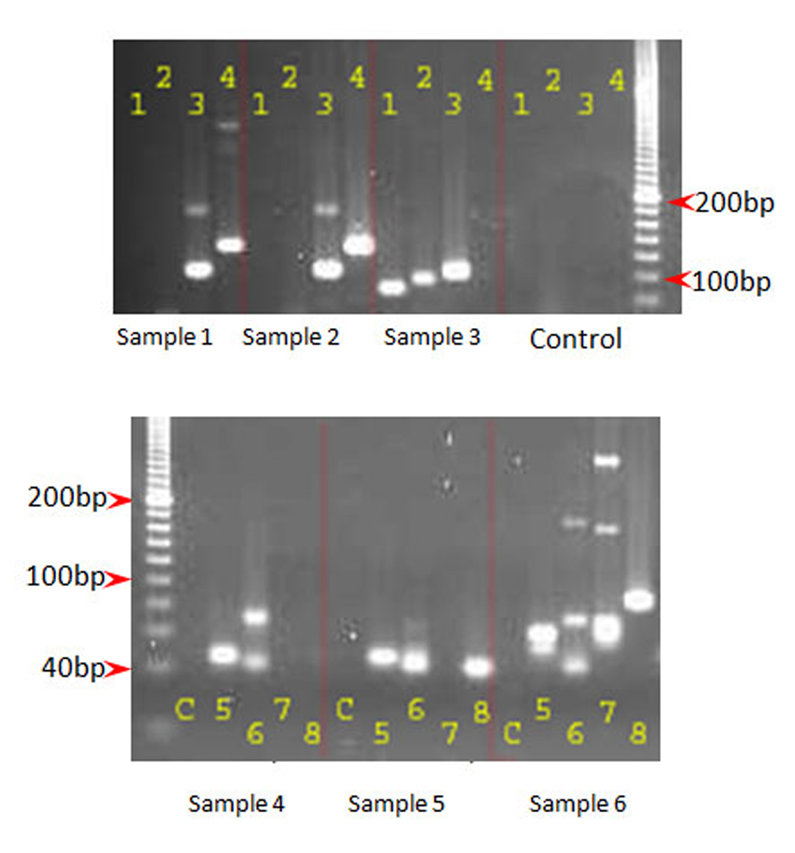

Supplement: S3 Fig — The type 1 variant is T1G2, indicating that exon1 of the TMPRSS2 gene (5’ of fused gene) is fused to exon2 of the ERG gene. Based on the same naming logic, the other seven variants are type 2(T1G3), type 3 (T1G4), type 4 (T1G5), type 5 (T2G2), type 6 (T2G4), type 7 (T2G5), and type 8 (T3G4). For each tissue sample, a separate RT-PCR was performed to amplify each variant. Negative control samples are denoted as C. Ladder was Biorad 20-bp DNA ladder. (TIF) [file pgen.1006477.s003.tif]

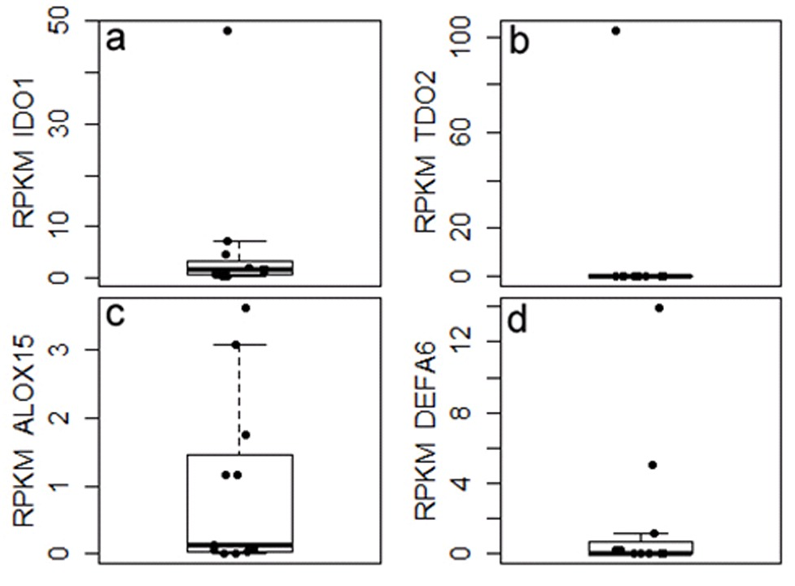

Supplement: S4 Fig — As examples, this figure displays 10 samples with outlying expression validated by RNAseq, including one sample for IDO1, one sample for TDO2, five samples for ALOX15, and three samples for DEFA6. (TIF) [file pgen.1006477.s004.tif]

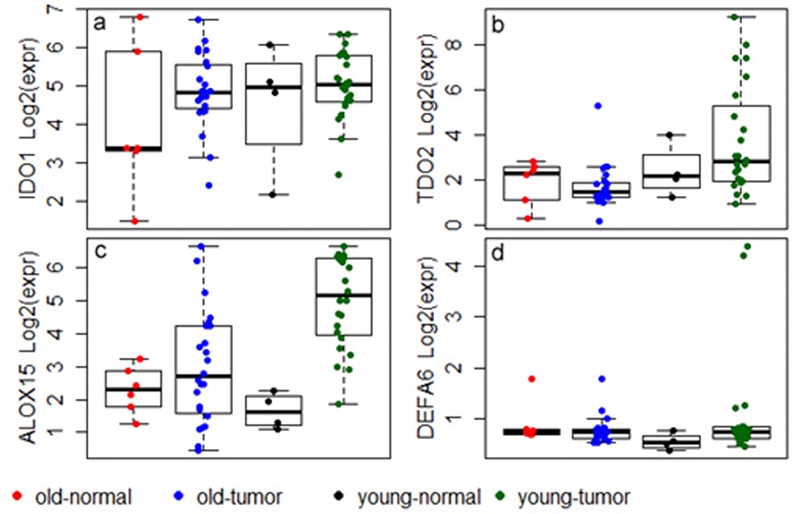

Supplement: S5 Fig — (TIF) [file pgen.1006477.s005.tif]

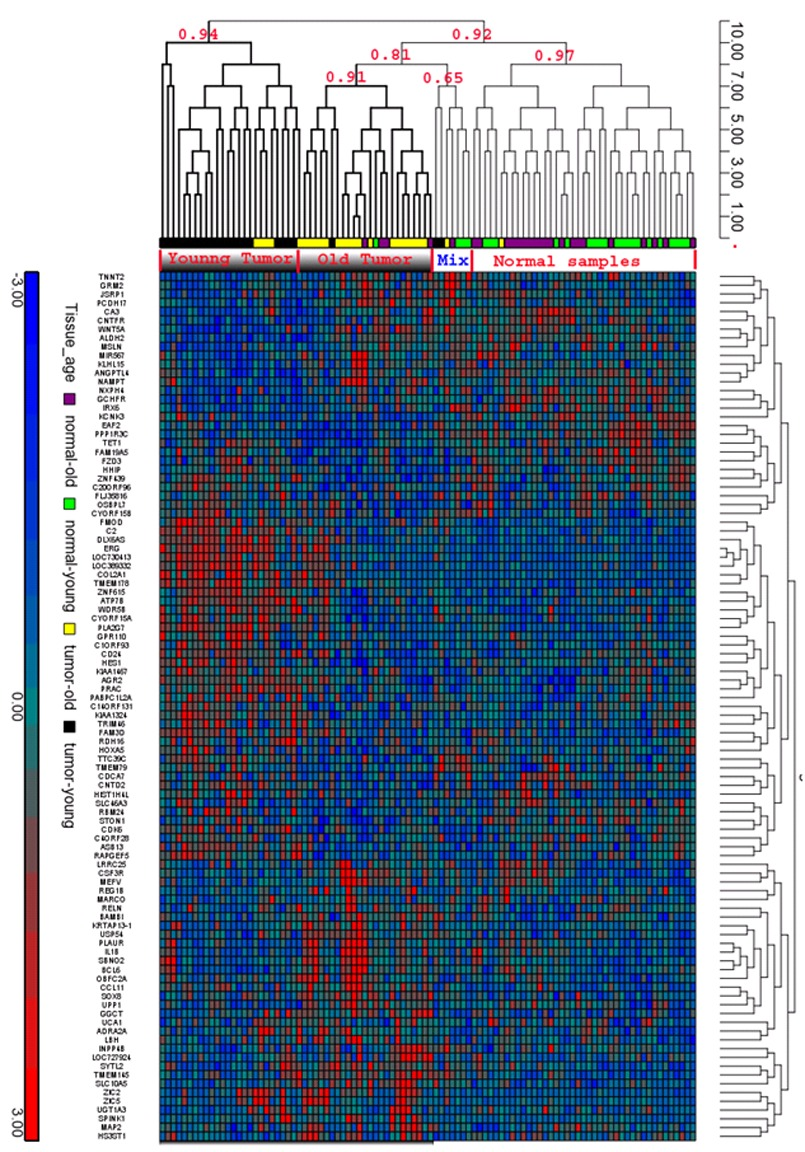

Supplement: S6 Fig — In clusters, columns are samples and rows are the genes. In the heat map, red corresponds to high expression, blue corresponds to low expression, and green corresponds to intermediate expression levels. (TIF) [file pgen.1006477.s006.tif]

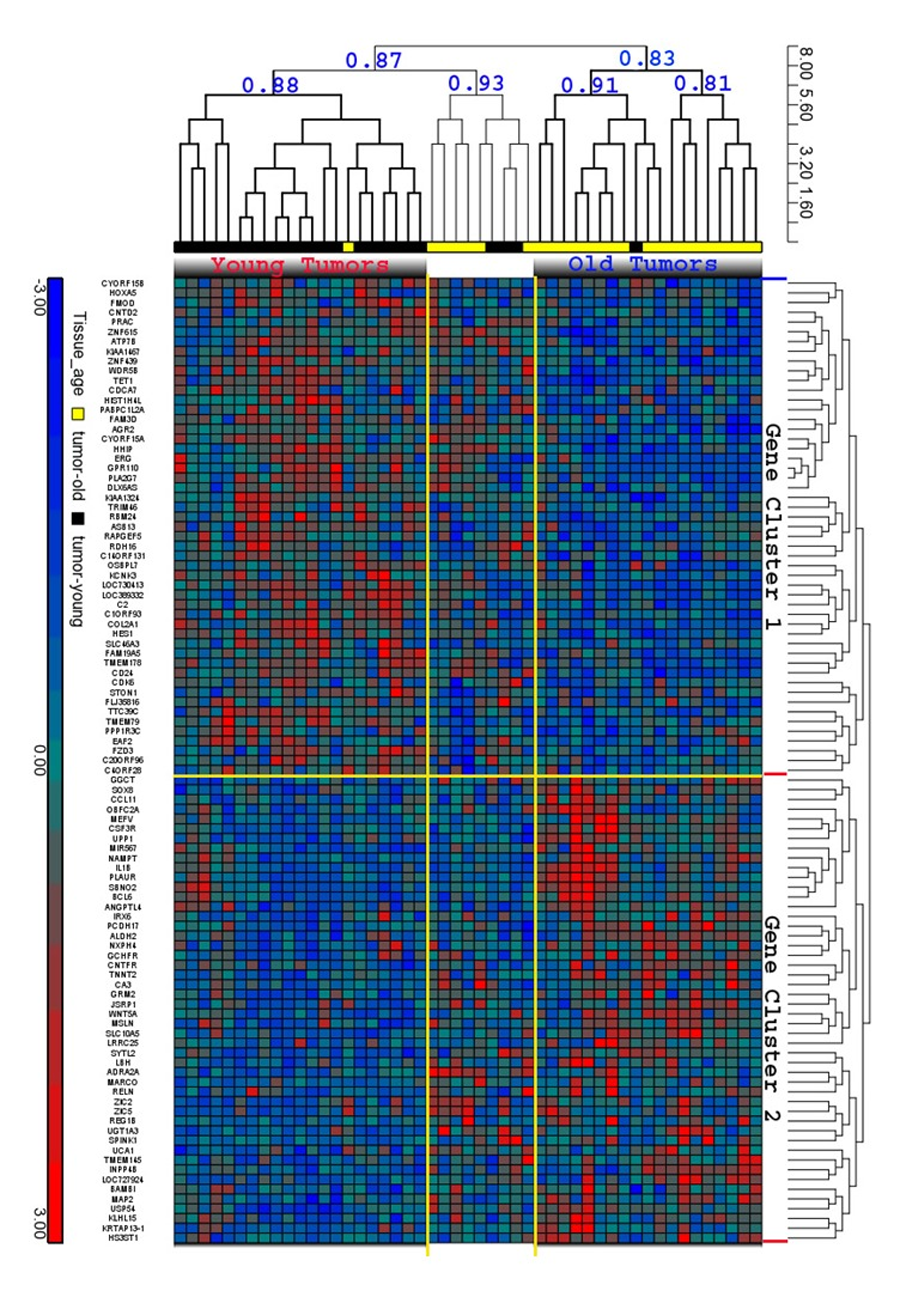

Supplement: S7 Fig — In clusters, columns are samples and rows are the genes. In the heat map, red corresponds to high expression, blue corresponds to low expression, and green corresponds to intermediate expression levels. (TIF) [file pgen.1006477.s007.tif]

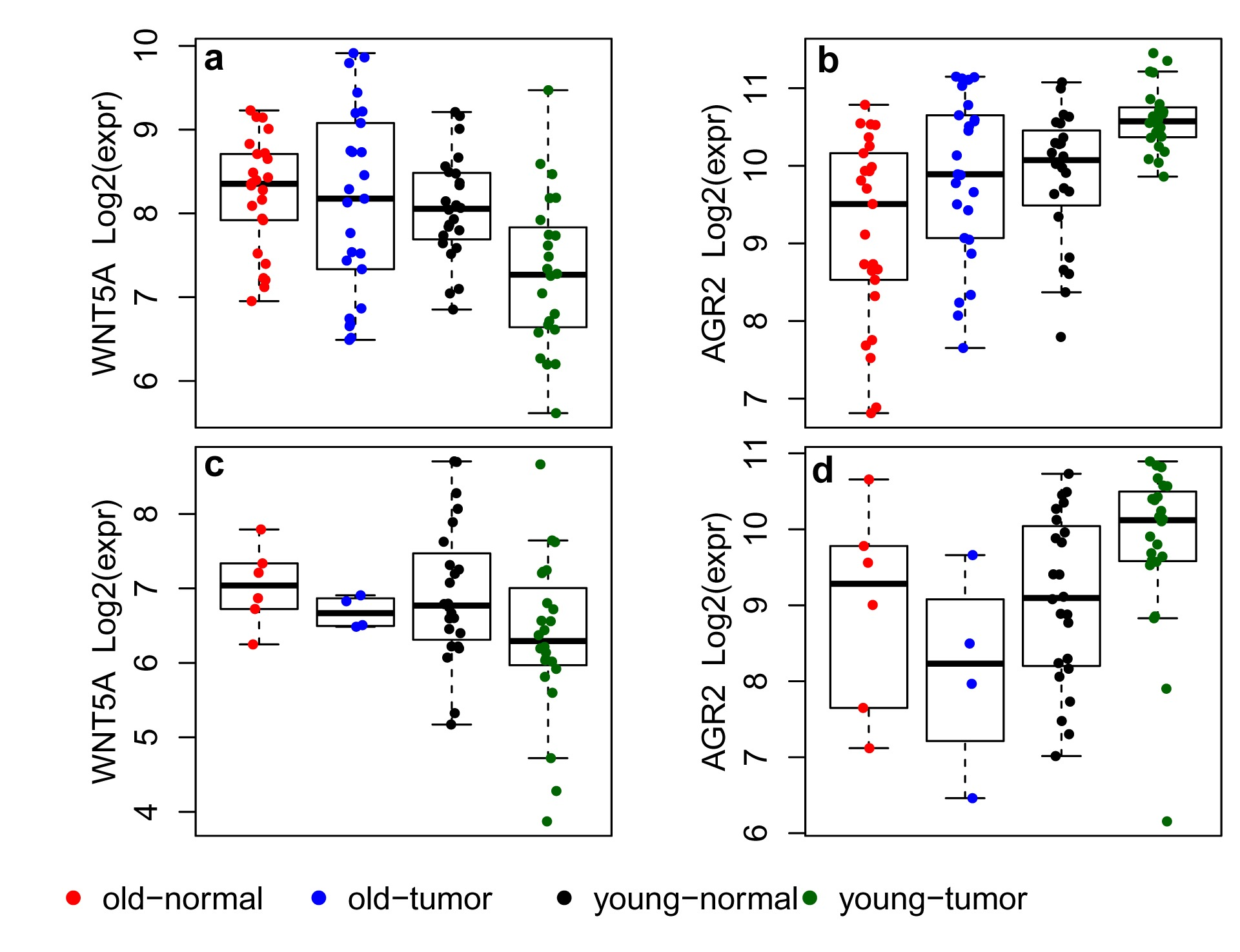

Supplement: S8 Fig — (TIF) [file pgen.1006477.s008.tif]

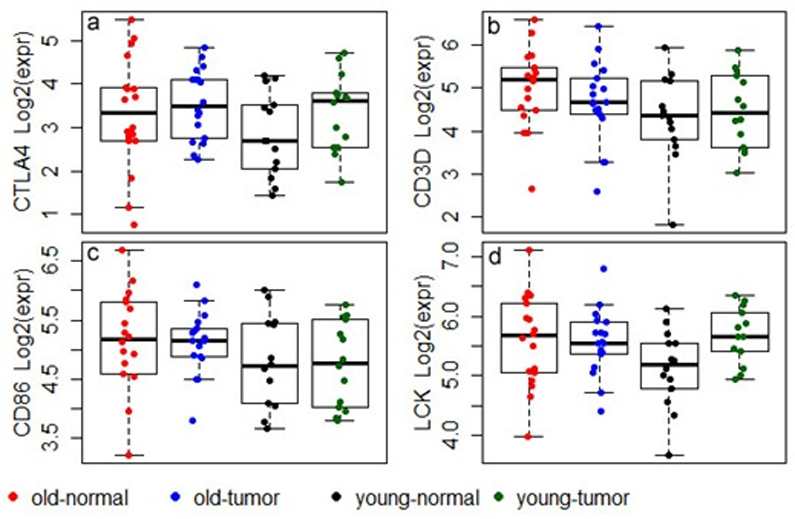

Supplement: S9 Fig — For TCGA data, mRNA expression data from paired normal tissue were available only for four young prostate cancer patients (≤ 50 years). Therefore, in order to check expression changes for genes in the CTLA4 pathway, we relaxed the age criteria to be older than 65 years (18 patients) and younger than 55 years (14 patients). This is consistent with the expression pattern in the DASL data for the CTLA4 and LCK genes (see Fig 1). (TIF) [file pgen.1006477.s009.tif]

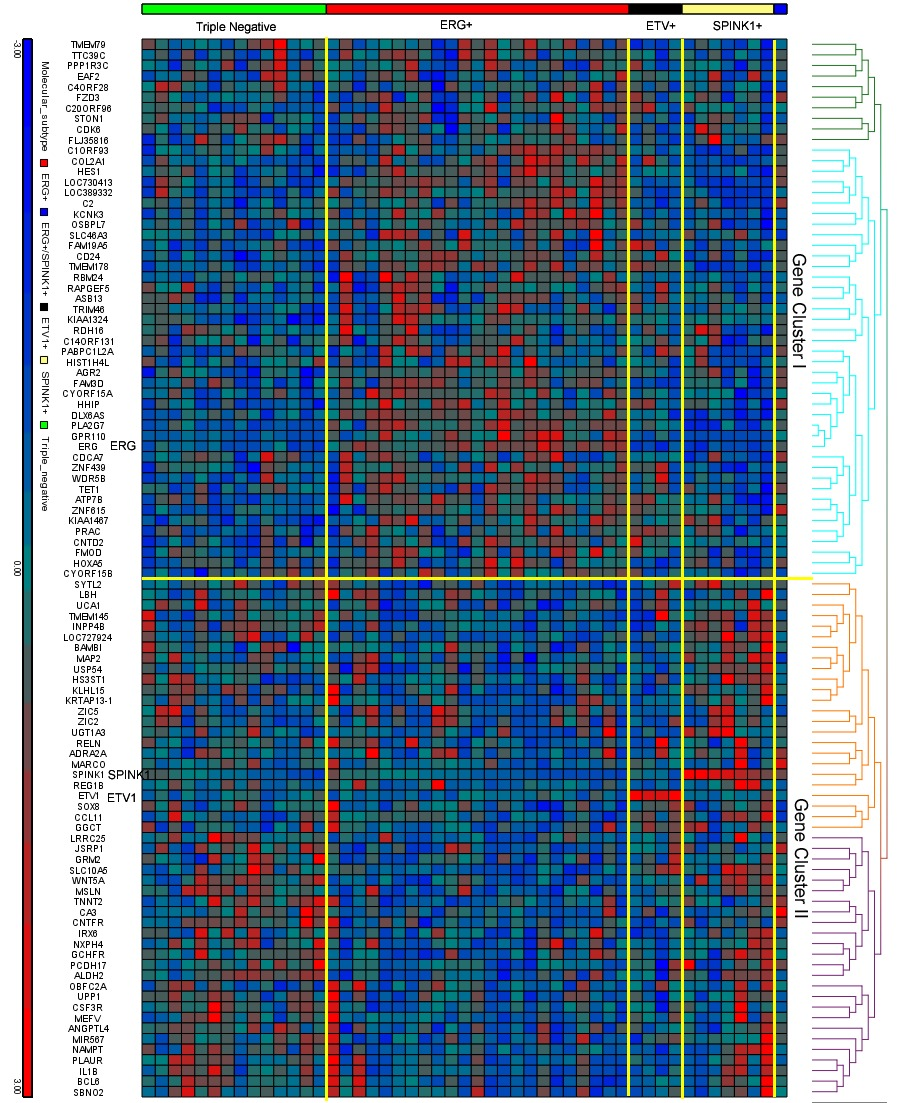

Supplement: S10 Fig — Tumor subtypes for the 49 tumor samples were assigned based on the DASL expression data of ERG, ETS, and SPINK1. Supervised hierarchical cluster analysis indicated that this set of genes cluster with known prostate subtypes. (TIF) [file pgen.1006477.s010.tif]
