# Supplementary material for: Gene Expression Differences in Prostate Cancers between Young and Old Men
Source: PLoS Genet. 2016 Dec 27;12(12):e1006477. doi: 10.1371/journal.pgen.1006477 (PMC5189936; doi:10.1371/journal.pgen.1006477)
Supplement: S1 Table — (DOCX) [file pgen.1006477.s011.docx]

S1 Table. Top-five Ingenuity Pathway Analysis (IPA) results for 62 of 183 DEGs (down-regulated in young compared to older cohort) from the age:tissue interaction contrast.

| **Top five Canonical Pathways** | **p-value** | **Overlap** |
| --- | --- | --- |
| Bupropion Degradation | 2.30E-03 | 8.0% (2/25) |
| Acetone Degradation I | 2.49E-03 | 7.7% (2/26) |
| Estrogen Biosynthesis | 5.00E-03 | 5.4% (2/37) |
| Spermine Biosynthesis | 5.70E-03 | 50.0% (1/2) |
| Spermidine Biosynthesis I | 8.54E-03 | 33.3% (1/3) |
|  |  |  |
| **Top Upstream Regulators** | **Activation z score** | **Predicted Activity** |
| SCAP, a gene with sterolsensing domain | -2.24 | Inhibited |
| SREBF2, Sterol Regulatory ElementBinding Transcription Factor 2 | -2.22 | Inhibited |
| EGR2, a transcription regulator | -2.00 | Inhibited |
|  |  |  |
| **Top five Molecular and Cellular Functions** | **p-value range** | **#Molecules** |
| Cellular Development | 2.82E-02-8.01E-06 | 24 |
| Lipid Metabolism | 2.82E-02-2.40E-05 | 14 |
| Small Molecule Biochemistry | 2.84E-02-2.40E-05 | 20 |
| Carbohydrate Metabolism | 1.70E-02-4.79E-05 | 7 |
| Molecular Transport | 2.82E-02-3.55E-04 | 15 |
