# Supplementary material for: Gene Expression Differences in Prostate Cancers between Young and Old Men
Source: PLoS Genet. 2016 Dec 27;12(12):e1006477. doi: 10.1371/journal.pgen.1006477 (PMC5189936; doi:10.1371/journal.pgen.1006477)
Supplement: S2 Table — (DOCX) [file pgen.1006477.s012.docx]

S2 Table. 21 of 62 down-regulated DEGs from age:tissue interaction contrast classified in the metabolic pathways.

| gene | interaction pattern* | P | FDR | FC | Gene annotation |
| --- | --- | --- | --- | --- | --- |
| ALDOC | c | 0.001 | 0.145 | -1.5 | aldolase C, fructose-bisphosphate |
| APLN | d | 0.000 | 0.116 | -1.5 | apelin |
| CARTPT | c | 0.001 | 0.170 | -2.1 | CART prepropeptide |
| CRYM | d | 0.001 | 0.133 | -1.5 | crystallin, mu |
| CYP1A2 | d | 0.001 | 0.164 | -1.5 | cytochrome P450, family 1, subfamily A, polypeptide 2 |
| CYP3A43 | c | 0.000 | 0.116 | -1.5 | cytochrome P450, family 3, subfamily A, polypeptide 43 |
| CYP51A1 | d | 0.004 | 0.208 | -1.5 | cytochrome P450, family 51, subfamily A, polypeptide 1 |
| ELOVL2 | g | 0.004 | 0.219 | -1.6 | elongation of very long chain fatty acids -like 2 |
| ELOVL7 | c | 0.000 | 0.096 | -1.6 | elongation of long chain fatty acids |
| GPR88 | d | 0.001 | 0.150 | -1.6 | G protein-coupled receptor 88 |
| INSIG1 | c | 0.000 | 0.116 | -1.7 | insulin induced gene 1 |
| NAMPT | d | 0.004 | 0.211 | -1.5 | nicotinamide phosphoribosyltransferase |
| NXPH4 | c | 0.006 | 0.235 | -1.5 | neurexophilin 4 |
| P2RY1 | c | 0.003 | 0.197 | -1.9 | purinergic receptor P2Y, G-protein coupled, 1 |
| PLP1 | c | 0.000 | 0.101 | -2.2 | proteolipid protein 1 |
| RELN | d | 0.000 | 0.052 | -2.2 | reelin |
| SCD | d | 0.001 | 0.150 | -1.6 | stearoyl-CoA desaturase |
| SMS | d | 0.001 | 0.170 | -1.5 | spermine synthase |
| SNCA | d | 0.001 | 0.144 | -1.5 | synuclein, alpha |
| ST6GALNAC5 | c | 0.000 | 0.116 |  |  |
| ZC3H12A | c | 0.007 | 0.247 | -1.6 | zinc finger CCCH-type containing 12A |

*age-tissue interaction pattern illustrated in Figure 1
