# Supplementary material for: Gene Expression Differences in Prostate Cancers between Young and Old Men
Source: PLoS Genet. 2016 Dec 27;12(12):e1006477. doi: 10.1371/journal.pgen.1006477 (PMC5189936; doi:10.1371/journal.pgen.1006477)
Supplement: S3 Table — (DOCX) [file pgen.1006477.s013.docx]

S3 Table. Gene Set Enrichment Analysis (GSEA) of down-regulated gene sets or pathways enriched in age-related differentially expressed genes in young compared to older cohorts.

| Top 10 gene sets or pathways ranked by Normalized Enrichment Score (NES) | Size | NES | FDR q-val |
| --- | --- | --- | --- |
| BIOCARTA_PROTEASOME_PATHWAY | 19 | -1.78 | 0.16 |
| **BIOCARTA_NOS1_PATHWAY** | 16 | -1.69 | 0.19 |
|  |  |  |  |
| KEGG_RNA_POLYMERASE | 27 | -2.11 | 0.00 |
| **KEGG_VALINE_LEUCINE_&_ISOLEUCINE_DEGRADATION** | 38 | -2.00 | 0.00 |
| **KEGG_OXIDATIVE_PHOSPHORYLATION** | 92 | -1.93 | 0.00 |
| **KEGG_FRUCTOSE_AND_MANNOSE_METABOLISM** | 28 | -1.91 | 0.00 |
| **KEGG_BIOSYNTHESIS_OF_UNSATURATED_FATTY_ACIDS** | 15 | -1.84 | 0.02 |
| **REACTOME_CHOLESTEROL_BIOSYNTHESIS** | 17 | -2.37 | 0.00 |
| REACTOME_RNA_POL_III_TRANSCRIPTION_INITIATION_TYPE_2_PROMOTER | 22 | -2.13 | 0.00 |
| **REACTOME_REGULATION_ORNITHINE_DECARBOXYLASE_ODC** | 35 | -1.97 | 0.03 |
| REACTOME_RNA_POL_III_TRANSCRIPTION_INITIATION_TYPE_3_PROMOTER | 24 | -1.97 | 0.03 |
| REACTOME_RNA_POL_III_CHAIN_ELONGATION | 16 | -1.94 | 0.03 |
| *Bold gene sets are related to metabolic function |  |  |  |
|  |  |  |  |
