# Supplementary material for: Gene Expression Differences in Prostate Cancers between Young and Old Men
Source: PLoS Genet. 2016 Dec 27;12(12):e1006477. doi: 10.1371/journal.pgen.1006477 (PMC5189936; doi:10.1371/journal.pgen.1006477)
Supplement: S4 Table — (DOCX) [file pgen.1006477.s014.docx]

S4 Table. Top-five IPA results for 79 genes with outliers of expression identified by the Cancer Outlier Profile Analysis (COPA).

| **Top Canonical Pathways** | | **p-value of overlap** | | **Overlap** |
| --- | --- | --- | --- | --- |
| 1. Tryptophan Degradation to 2-amino-3 carboxymuconate Semialdehyde | | 3.08E-04 | 25.0 % (2/8) | |
| 2. Hepatic Fibrosis / Hepatic Stellate Cell Activation | | 3.79E-04 | 2.7% (5/183) | |
| 3. NAD biosynthesis II (from tryptophan) | | 1.14E-03 | 13.3% (2/15) | |
| 4. Tryptophan Degradation III (Eukaryotic) | 2.25E-03 | | 9.5% (2/21) | |
| 5. Role of Macrophages, Fibroblasts & Endothelia Cells in Rheumatoid Arthritis | 3.21E-03 | | 1.7% (5/296) | |
|  |  | |  | |
| **Top Upstream Regulators** | **p-value of overlap** | | **Target molecules in dataset** | |
| 1. RAR ligand-RARα-Retinoic acid-RXRα | 5.58E-05 | | SLCO1A2,SLCO1B3 | |
| 2. Interferon γ receptor-deficient | 1.51E-04 | | AGT,NOS2 | |
| 3. STAT1 Transcription factor | 1.73E-04 | | AGT,ALOX15,IDO1,NOS2 | |
| 4. Toll like receptors, Tlr | 4.11E-04 | | IDO1,NOS2,SPHK1 | |
| 5. NFkB (complex) | 4.40E-04 | | AGT,COL2A1,IDO1  MMP1,NOS2,SLC3A1 | |
|  |  | |  | |
|  | | | | |
| **Top Diseases and Disorders** | **p-value range** | | **number of Molecules** | |
| 1. Inflammatory Response | 6.72E-03 - 9.79E-06 | | 5 | |
| 2. Cardiovascular Disease | 6.72E-03 - 1.29E-05 | | 10 | |
| 3. Dermatological Diseases and Conditions  (Chronic large plague psoriasis) | 6.72E-03 - 1.29E-05 | | 42 | |
| 4. Organismal Injury and Abnormalities | 6.72E-03 - 1.29E-05 | | 66 | |
| 5. Cancer | 6.72E-03 - 4.56E-05 | | 65 | |
