# Supplementary material for: Gene Expression Differences in Prostate Cancers between Young and Old Men
Source: PLoS Genet. 2016 Dec 27;12(12):e1006477. doi: 10.1371/journal.pgen.1006477 (PMC5189936; doi:10.1371/journal.pgen.1006477)
Supplement: S5 Table — (DOCX) [file pgen.1006477.s015.docx]

S5 Table. A flow diagram for selection of samples from TCGA.

|  |  |  | 545 TCGA prostate samples |  |
| --- | --- | --- | --- | --- |
|  |  |  | **↓** |  |
|  |  |  | 96 samples with age <50 (young) or age > 70 (old) |  |
|  |  |  | **↓** |  |
|  |  |  | 85 samples after removing 11 normal samples |  |
|  |  |  | **↓** |  |
|  |  |  | 81 remaining samples after removing 3 samples with pathology stage T3 and 1 with Gleason 10 |  |
|  |  |  | **↓** |  |
|  |  |  | 48 samples selected (24 from older men and 24 from young men) with matched tumor stage and Gleason between the two age groups using random numbers |  |
|  |  |  | **↓** |  |
| Gleason_sum | # of young samples | # of old samples | Samples selection | Final  included samples |
| 6 | 7 | 3 | randomly select 3 of 7 young samples using random numbers | 6 |
| 7 | 14 | 9 | random select 9 of 14 young samples using random numbers | 18 |
| 8 | 2 | 4 | random select 2 of 4 old samples using random numbers | 4 |
| 9 | 0 | 1 | no samples included for samples with T2 Gleason 9 | 0 |
| 6 | 0 | 1 | no samples included for samples with T3 Gleason 6 | 0 |
| 7 | 5 | 10 | random select 5 of 10 old samples using random numbers | 10 |
| 8 | 2 | 5 | random select 2 of 5 old samples using random numbers | 4 |
| 9 | 3 | 15 | random select 3 of 15 old samples using random numbers | 6 |
|  | 33 | 48 |  | 48 |
