# Supplementary material for: Gene Expression Differences in Prostate Cancers between Young and Old Men
Source: PLoS Genet. 2016 Dec 27;12(12):e1006477. doi: 10.1371/journal.pgen.1006477 (PMC5189936; doi:10.1371/journal.pgen.1006477)
Supplement: S6 Table — (DOCX) [file pgen.1006477.s016.docx]

S6 table. Clinical characteristics of 48 TCGA samples.

|  | Total (N=48) | old (N=24) | young (N=24) |
| --- | --- | --- | --- |
| Age range in years |  | 70-77 | 43-50 |
|  |  |  |  |
|  | N (%) | N (%) | N (%) |
| Pathology stage |  |  |  |
| T2 | 28 (58) | 14 (58) | 14 (58) |
| T3 | 20 (42) | 10 (42) | 10 (42) |
|  |  |  |  |
| Gleason sum |  |  |  |
| 6 | 6 (12) | 3 (12) | 3 (12) |
| 7 | 28 (58) | 14 (58) | 14 (58) |
| 8 | 8 (17) | 4 (17) | 4 (17) |
| 9 | 6 (13) | 3 (13) | 3 (13) |
|  |  |  |  |
| Race/ethnicity |  |  |  |
| Whites | 16 (33) | 8 (33) | 8 (33) |
| African Americans |  |  |  |
| Hispanics |  |  |  |
| Asians |  |  |  |
| Unknown | 32 (67) | 16 (67) | 16 (67) |
